# The Quinovic Acid Glycosides Purified Fraction from *Uncaria tomentosa* Protects against Hemorrhagic Cystitis Induced by Cyclophosphamide in Mice

**DOI:** 10.1371/journal.pone.0131882

**Published:** 2015-07-08

**Authors:** Fabrícia Dietrich, Jerônimo Pietrobon Martins, Samuel Kaiser, Rodrigo Braccini Madeira Silva, Liliana Rockenbach, Maria Isabel Albano Edelweiss, George González Ortega, Fernanda Bueno Morrone, Maria Martha Campos, Ana Maria Oliveira Battastini

**Affiliations:** 1 Programa de Pós-Graduação em Ciências Biológicas: Bioquímica, ICBS, UFRGS, Porto Alegre, RS, Brazil; 2 Departamento de Bioquímica, ICBS, UFRGS, Porto Alegre, RS, Brazil; 3 Laboratório de Farmacologia Aplicada, Faculdade de Farmácia, PUCRS, Porto Alegre, RS, Brazil; 4 Programa de Pós-Graduação em Ciências Farmacêuticas, Faculdade de Farmácia, UFRGS, Porto Alegre, RS, Brazil; 5 Instituto de Toxicologia e Farmacologia, PUCRS, Porto Alegre, RS, Brazil; 6 Faculdade de Medicina, UFRGS, Porto Alegre, RS, Brazil; 7 Faculdade de Odontologia, PUCRS, Porto Alegre, RS, Brazil; University of South Carolina School of Medicine, UNITED STATES

## Abstract

*Uncaria tomentosa* is widely used in folk medicine for the treatment of numerous diseases, such as urinary tract disease. Hemorrhagic cystitis (HE) is an inflammatory condition of the bladder associated with the use of anticancer drugs such as cyclophosphamide (CYP). Sodium 2-mercaptoethanesulfonate (Mesna) has been used to prevent the occurrence of HE, although this compound is not effective in established lesions. It has been demonstrated that the purinergic system is involved in several pathophysiological events. Among purinergic receptors, P2X7 deserves attention because it is involved in HE induced by CYP and, therefore, can be considered a therapeutic target. The objective of this study was to investigate the potential therapeutic effect of the quinovic acid glycosides purified fraction (QAPF) from *U*. *tomentosa* in the mouse model of CYP-induced HE. Pretreatment with QAPF not only had a protective effect on HE-induced urothelial damage (edema, hemorrhage and bladder wet weight) but was also able to control visceral pain, decrease IL-1β levels and down-regulates P2X7 receptors, most likely by inhibit the neutrophils migration to the bladder. This research clearly demonstrates the promising anti-inflammatory properties of QAPF, supporting its use as complementary therapy. QAPF represents a promising therapeutic option for this pathological condition.

## Introduction

*Uncaria tomentosa* (Willd.) DC. belongs to the Rubiaceae family, commonly known as “uña de gato” or “cat's claw” due to its curved hooks [1]. For centuries, decoctions of its bark and roots have been used by the Asháninkas people [1, 2, 3]. Particularly in traditional Peruvian medicine, preparations of *U*. *tomentosa* have been used for the treatment of gastric ulcers, arthritis, viral infections, general inflammatory conditions, urinary tract disease, and cancer [1, 3]. In addition, dietary supplements obtained from *U*. *tomentosa* bark have been widely used as adjunctive therapies for the treatment of inflammatory diseases [4, 5]. The wide range of activities conferred to *U*. *tomentosa* is mostly attributed to the presence of three main fractions of secondary metabolites: polyphenols, alkaloids and quinovic acid glycosides [3]. *U*. *tomentosa* decoctions have demonstrated an anti-inflammatory potential by preventing or modulating lung injury induced by ozone in vivo [6]. For example, hydroalcoholic extracts of *U*. *tomentosa* and quinovic acid glycoside were able to decrease carrageenan-induced paw edema in mice and rats, respectively [7, 8]. Although the mechanisms by which *U*. *tomentosa* exerts anti-inflammatory activity remain unclear, its biological actions are likely related to the modulation of TNF synthesis, via NF-κB inhibition [2, 9, 10, 11]. However, no study has evaluated the anti-inflammatory effect of *U*. *tomentosa* against hemorrhagic cystitis (HE).

HE is a general inflammatory condition of the urinary bladder [12] characterized by intense pain, edema, bleeding, ulceration, necrosis, and leukocyte infiltration [13]. HE is usually associated with the use of the anticancer drug cyclophosphamide (CYP) [12]. The incidence of HE is correlated with the CYP dosage and can be higher than 75% [14]. The urological side effects of CYP are mediated by the metabolite acrolein, which is able to induce severe toxicity by direct contact with the urothelium and by evoking a marked inflammatory reaction [12, 15]. Sodium 2-mercaptoethanesulfonate (Mesna) is prophylactically administered to patients treated with CYP because this compound binds to acrolein, reducing harm to the bladder [15]. However, Mesna has no effectiveness in established lesions, as morbidity due to HE continues to increase [13, 16, 17]. The involvement of purinergic signaling in the physiology of the urinary bladder has been extensively studied [18], with special focus on malignancies of the genitourinary tract [19]. Extracellular purines and pyrimidines exert different effects by interacting with purinoreceptors P1 and P2. The P2 receptors are divided into two families, P2Y and P2X [20]. Among the P2X receptors, P2X7 has demonstrated an important role in the cystitis hemorrhagic model induced by CYP. It was shown that the pharmacological blockade or genetic deletion of this receptor reduced the nociceptive and inflammatory events associated with HE [21]. Thus, the association of the P2X7 receptor (P2X7R) with inflammation observed in HE becomes a possible target for the treatment of this disease.

Therefore, considering the numerous pharmacological properties attributed to *U*. *tomentosa* and the results already obtained on the anti-inflammatory activity of this species, the present study aimed to investigate the potential therapeutic effect of the quinovic acid glycosides purified fraction (QAPF) obtained from *U*. *tomentosa* stem bark in a mouse model of CYP-induced HE. The involvement of the P2X7R in the anti-inflammatory effect of QAPF on HE induced by CYP was also investigated. In this study, QAPF from *U*. *tomentosa* reduces nociceptive and inflammatory events mediating your effects by a mechanism that involves the reduced migration of neutrophils, and as consequence of this, down-regulation of its P2X7R and the decrease of IL-1β levels in the urinary bladder. Thus, QAPF may be deemed promising for the prevention or treatment of this disease.

## Materials and Methods

### Chemicals

Cyclophosphamide (Genuxa1@200) and Mesna (Mitexan) were obtained from Baxter Oncology GmbH (Frankfurt, Germany). Dimethyl sulfoxide (DMSO), tetramethylbenzidine (TMB) and hexadecyltrimethylammonium bromide (HTAB) were obtained from Sigma Chemical Co. (St Louis, MO, USA). NaCl, NaPO_4_, hydrogen peroxide and Tween 20 were obtained from Merck (Haar, Germany). BSA Fraction V (7.5%) was obtained from Gibco (Grand Island, NY, USA). ELISA kits for quantifying cytokine levels were obtained from R&D Systems (Minneapolis, MN, USA). All other chemicals and solvents used were of analytical or pharmaceutical grade.

### Ethics statement

All experimental procedures used in the present study were performed in accordance with the Guidelines for the Use and Care of Laboratorial Animals from National Institute of Health publication No. 85–23 and ethical guidelines for investigations of experimental pain in conscious animal. The protocols were approved by the Ethics Committee of Pontifícia Universidade Católica do Rio Grande do Sul (CEUA 11/00243). The number of animals and the intensity of noxious stimuli were the minimum required to demonstrate consistent effects of the drug treatment.

### Animals

Male Swiss mice (25 to 30 g) were housed in groups of five per cage and kept under conditions of optimum light (12 h light/dark cycle), controlled temperature (22 ± 2°C) and humidity (60–70%), with food and water *ad libitum*. Except in behavioral studies, the experiments were performed between 8:00 AM and 6:00 PM. At least 1 h before the start of the experiments, the animals were acclimatized in the laboratory. The number of animals used in each experiment is provided in the legend of the figures.

### Obtaining the purified fraction

Stem bark of *U*. *tomentosa*, supplied by Laboratorios Induquímica S.A., (Lima, Peru) collected in Ucayali (Peru) and botanically certified by Peruvian biologist José Ricardo Campos de La Cruz (Universidad Nacional Mayor de San Marcos, Lima, Peru), were used in the present study. The quinovic acid glycosides purified fraction (QAPF) was obtained and suitably characterized by HPLC-PDA and UPLC/Q-TOF-MS analysis, as previously described [22, 23]. All samples were properly concentrated and freeze-dried.

### Hemorrhagic cystitis induction and dose treatment schedule

Hemorrhagic cystitis was induced by a single intraperitoneal (i.p.) injection of CYP (300 mg/kg) [21]. The animals received two administrations of the following substances: the reference compound Mesna (60 mg/kg, i.p.) or QAPF (20, 50 and 100 mg/kg, i.p.); the first dose was given 30 min prior to CYP, and the subsequent dose was administered 4 h after the injection of CYP, except in the experiments for assessing cytokines, in which the drugs were administered as a single i.p. dose 30 min prior to CYP. CYP and Mesna were diluted in 0.9% NaCl. The purified fraction from *U*. *tomentosa* was prepared in 3% DMSO diluted with sterile water. The control groups received vehicle solutions: 3% DMSO plus saline (control group) or purified fraction plus saline (drug control) at the same schedules of administration (Table 1). The doses of the fraction were chosen on the basis of literature data [1, 8, 24]. Each group consisted of at least three animals.

**Table 1:** Detailed scheme of the treatment groups.

Group	Dose (mg/kg)	Dose schedule	Administration route
**DMSO 3% + Saline**	—	30 min prior; 4 h after Saline/ at 30 min	i.p.^a^
**DMSO 3% + CYP** ^**b**^	— + 300	30 min prior; 4 h after CYP/ at 30 min	i.p.
**QAPF** ^**c**^ **+ Saline**	20 or 100 +—	30 min prior; 4 h after Saline/ at 30 min	i.p.
**Mesna** ^**d**^ **+ CYP**	60 + 300	30 min prior; 4 h after CYP/ at 30 min	i.p.
**QAPF + CYP**	20, 50 or 100 + 300	30 min prior; 4 h after CYP/ at 30 min	i.p.

Detailed scheme of hemorrhagic cystitis induction and treatment schedule of the groups.

^a^i.p. = intraperitoneal

^b^CYP = cyclophosphamide

^c^QAPF = quinovic acid glycosides purified fraction

^d^Mesna = Sodium 2-mercaptoethanesulfonate

The animals were killed by cervical dislocation 6 h following CYP administration, and the bladders were collected for macroscopic and histological analyses, as described below.

### Behavioral studies–nociceptive response

To minimize the potential circadian variations in behavioral responses, the experiments were performed between 8:00 and 12:00 AM. Immediately after i.p. CYP administration, mice were housed in individual plastic cages without sawdust, and the spontaneous behavior was measured for 2 min, every 30 min, during a total period of 4 h. The following behavioral changes were evaluated: (i) activity (walking, rearing, climbing, grooming); (ii) immobility; and (iii) behaviors indicative of visceral pain (‘crises’). In addition, the behavioral alterations were scored according to the following scale: 0 = normal; 1 = piloerection; 2 = strong piloerection; 3 = labored breathing; 4 = licking of the abdomen; or 5 = stretching and contractions of the abdomen [21]. The nociceptive responses were provided as the sum of the total score.

### Assessment of visceral sensitivity using von Frey filaments

Hyperalgesia is characterized by an enhanced abdominal sensitivity due to irritation and/or inflammation in the pelvic viscera and is evaluated by mechanical stimulation with von Frey filaments on the lower abdominal/pelvic area [25]. The animals were placed individually in chambers with a wire mesh floor (mouse acclimation period was 30 min before behavioral testing). Mice were tested one day before (baseline) and 1.5 h after the HE induction. Frequency of withdrawal responses was tested using a 0.4 g von Frey monofilament (Stoelting, USA). The filament was applied vertically on the lower abdominal/pelvic area on mice with a pressure high enough to bend the filament. Some criteria were described as follows: i) lower abdominal/pelvic area withdrawal was considered when the animal completely removed the area; ii) each animal was stimulated for 1–2 s for a total of 10 applications; iii) each withdrawal was recorded as 10% of response; and iv) stimulation was confined to the lower abdominal area in the general vicinity of the bladder. Different areas within this region were stimulated to avoid desensitization.

### Bladder gross evaluation and wet weight determination

The animals were killed 6 h following CYP administration, and the gross evaluation of the bladder was based on criteria previously established [26]. All bladders were dissected free of connective tissues and transected at the bladder neck. An examiner blind to the experimental groups macroscopically evaluated each bladder. The bladders were evaluated for bleeding in the walls and were categorized into four designations considering (3) the presence of intravesical clots; (2) mucosal hematomas; (1) telangiectasia or dilatation of the bladder vessels; and (0) normal aspect. The edema formation was classified as (3) severe, when fluid was observed externally and internally in the walls of the bladder; (2) moderate, when the edema was confined to the internal mucosa; (1) mild, when slight edematogenic signals were observed; and (0) absent, when the bladder was normal. As an additional measure of bladder edema, the wet weight of each bladder was recorded and expressed as grams per 100 g of animal.

### Histological analysis

After 6 h of treatment, the animals were killed, and the bladders were removed and fixed in buffered formalin solution (10%) for 24 h. Subsequently, the fixed tissue samples embedded in paraffin were stained with hematoxylin and eosin, and a pathologist performed the pathological analysis of the slides in a blinded manner. As proposed by Gray *et al*. [26], the following parameters were considered: normal (normal urothelium, no inflammatory cell infiltrate or ulcers); mild (diminished urothelial cells, flattening with submucosal edema, mild hemorrhage, few ulcerations); moderate (mucosal erosion, inflammatory cell infiltrate, fibrin deposition, hemorrhage, and multiple ulcerations); and severe (mucosal erosion, inflammatory cell infiltrate, fibrin deposition, multiple ulcerations, and transmural hemorrhage with severe edema). In addition, the histological alterations were scored according to the following scale: 1 = normal urothelium; 2 = diminished urothelial cells; 1 = no inflammatory cell infiltrate; 2 = presence of inflammatory cell infiltrate; 1 = no ulcers; 2 = few ulcerations; 3 = multiple ulcerations; 1 = no edema; 2 = mild edema; 3 = severe edema; 1 = no hemorrhage; 2 = mild hemorrhage; 3 = severe hemorrhage; 1 = no fibrin deposition; 2 = fibrin deposition. The maximum score to be obtained was 15.

### Myeloperoxidase (MPO) activity

Neutrophil recruitment to mouse bladder was measured by means of tissue MPO activity according to a reported method [27]. After 6 h of CYP administration, the bladders of Swiss mice were removed, homogenized (5% EDTA/NaCl buffer, pH 4.7) and centrifuged at 2700×*g* for 15 min at 4 °C. The pellet was resuspended in 0.5% HTAB buffer (pH 5.4), and the samples were centrifuged again; the supernatant (25 µL) was used in the MPO assay. The enzymatic reaction was assessed with 1.6 mM TMB, 80 mM NaPO_4_ and 0.3 mM H_2_O_2_. The absorbance was measured at 595 nm, and the results are expressed in optical density per grams of tissue.

### Determination of cytokine levels

Saline, Mesna and QAPF were administered to the animals as a single i.p. dose, 30 min prior to CYP. As the increase in IL-1β levels peaked 4 h following CYP administration, this time interval was adopted in this experiment. Therefore, after 4 h, the animals were killed, and their bladders were collected. Tissues were homogenized (PBS containing 0.05% Tween-20, 0.1 mM phenylmethylsulphonyl fluoride, 0.1 mM benzethonium chloride, 10 mM EDTA and 20 KIU aprotinin A), centrifuged at 3000×*g* for 10 min and stored at -70 °C for later measurement of IL-1β levels, according to the protocol described previously [21].

### Immunohistochemistry for P2X7 receptor

Expression of the P2X7 purinergic receptor in bladder tissue was evaluated by immunohistochemical analysis. For this experiment, the bladders were collected 6 h after hemorrhagic cystitis induction. The expression of P2X7 receptors was determined using the rabbit anti-P2X7 receptor (extracellular) polyclonal antibody (1:200; catalogue number APR-008, Alomone, Jerusalem, Israel). The secondary antibody used was rabbit anti-mouse secondary antibody (REVEAL Biotin-Free Polyvalent HRP; catalogue number SPB-999, Spring Bioscience Corporation, CA, USA). The bladder submucosal layer was selected with a region of interest and the mean of P2X7R-immunoreactive cells was acquired in number by Image J Software. Images were quantified by counting 10 pictures obtained of each slide per mouse, which was defined to be enough to capture almost all tissue. With the mean of P2X7R-immunoreactive cells, the statistical analysis comparing control, CYP and QAPF treatment was performed. These images were captured by optical microscope (Nikon Eclipse TE 300) and quantified by two people blinded to the study. Also, these images were analyzed by a pathologist blinded to the experimental data.

### Statistical analysis

The results were expressed as the mean ± SEM and were analyzed by one-way ANOVA followed by Tukey’s *post-hoc* test. Differences between groups were considered significant when *p*<0.05.

## Results

### QAPF decreases CYP-induced nociceptive behavior

**Fig 1:**
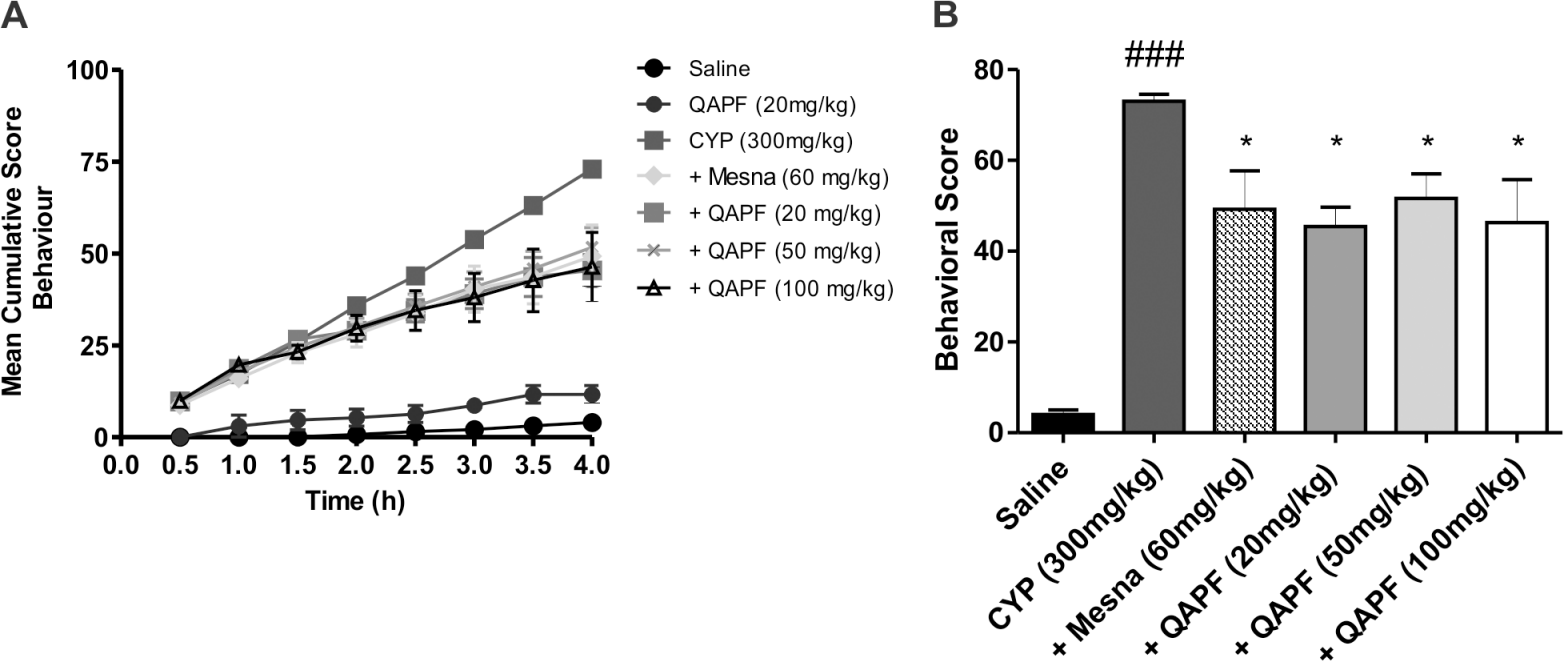
Behavioral scores, assigned at 30-min intervals, over 4 h after injection of cyclophosphamide. (A) Time-course of behavioral scores was plotted as the cumulative score assigned at 30-min intervals during 4 h, after the injection period of different treatments for the nociceptive response in CYP-induced hemorrhagic cystitis in mice. (B) Effect of treatment with Mesna or QAPF from *Uncaria tomentosa* on the nociceptive response after 4 h. The nociceptive responses were provided as described in Materials and Methods. Each column represents the mean of at least 5 animals, and the vertical lines show the SEM. ^###^ *p*<0.001 denotes the significance levels compared to saline values; ^*^ *p*<0.05 denotes the significance levels compared to CYP values. +Mesna represents CYP+Mesna; +QAPF represents CYP+QAPF.

Mice treated with CYP (300 mg/kg) displayed significant behavioral changes compared to control mice, which can be associated with development of pelvic pain related with CYP-induced HE (Fig 1). A reduction in CYP-induced nociceptive changes occurred with QAPF treatment throughout the evaluation, with an efficacy comparable to the reference compound Mesna (Fig 1A). It is important to note that QAPF-treatment caused a significant inhibition of the nociceptive responses near the end of the 4-h period (Fig 1B). QAPF alone (20 mg/kg) did not cause significant changes in the nociceptive behavioral score of the animals (Fig 1A).

**Fig 2:**
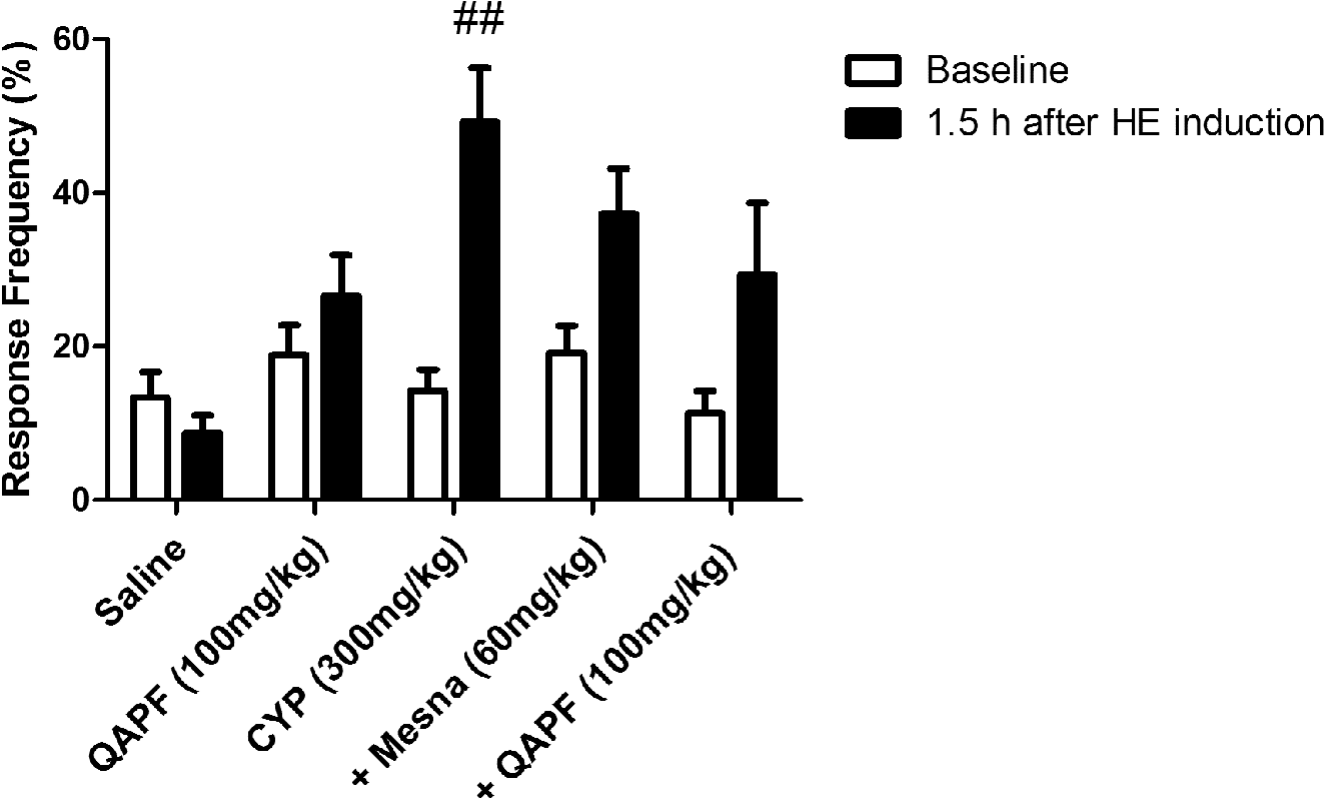
Effect of treatment with Mesna or QAPF from *Uncaria tomentosa* on the development of visceral sensitivity. The visceral sensitivity was measured by the frequency of responses to von Frey filament testing in the lower abdominal area one day before (baseline) and 1.5 h after the HE induction. Each column represents the mean of at least 8 animals, and the vertical lines show the SEM. ^##^ *p*<0.01 denotes the significance levels compared to saline values. +Mesna represents CYP+Mesna; +QAPF represents CYP+QAPF.

To confirm the effects of QAPF on nociception, we evaluated visceral sensitivity using von Frey filaments. Fig 2 summarizes the frequency of responses to von Frey filament stimulation of the lower abdominal area one day before and 1.5 h after HE induction. We observed that after the injection of CYP, mice became more sensitive to the filament and the response frequency reached almost 50% using a 0.4 g von Frey filament. Moreover, mice treated with QAPF presented a similar profile regarding behavior nociceptive score (Fig 1B) and visceral sensitivity (Fig 2) when compared to CYP-mice, suggesting that QAPF was able to inhibit hypersensitivity in the pelvic area, which may lead to reduced abdominal discomfort and/or pelvic pain. Furthermore, none of the treatments produced significant changes in locomotor parameters in the open-field arena (S1 Fig).

### QAPF decreases hemorrhage and edema caused by CYP

**Fig 3:**
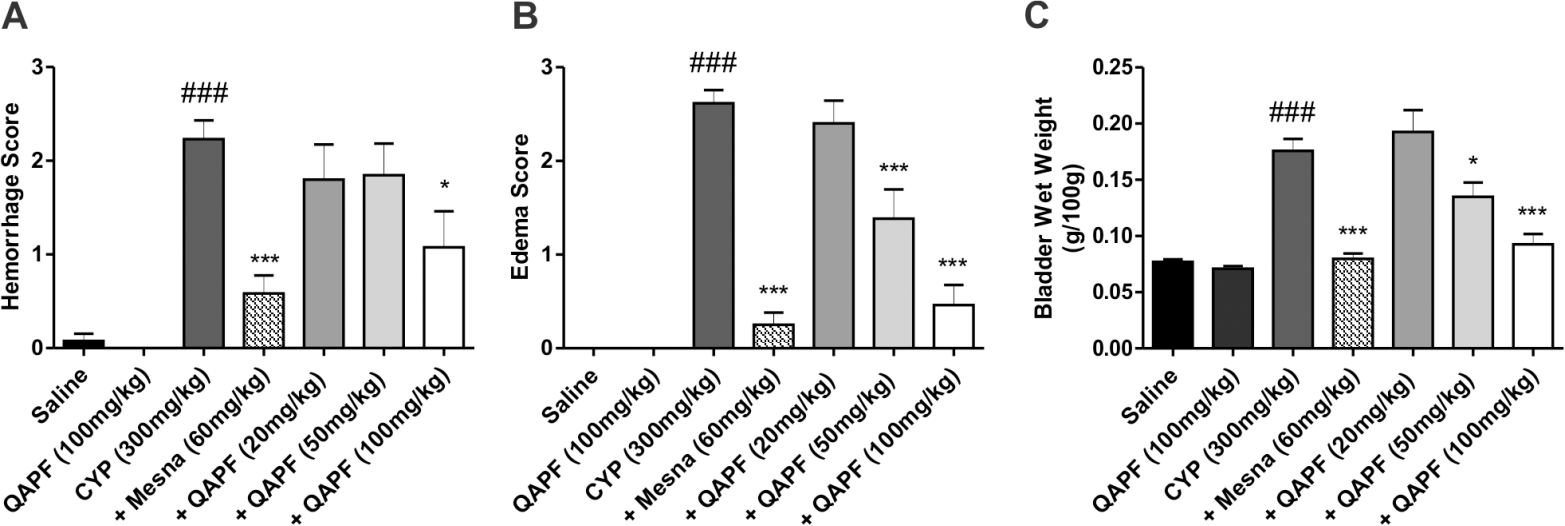
Effect of treatment with Mesna or QAPF from *Uncaria tomentosa* on macroscopic evaluation of the bladder. The (A) hemorrhage, (B) edema, and (C) wet bladder weight was evaluated in CYP-induced hemorrhagic cystitis in mice. In graphics A–C, each column represents the mean of 12–13 animals, except for +QAPF 20 mg/kg, in which five animals were used. The vertical lines show the SEM. ^###^ *p*<0.001 denotes the significance levels compared to saline values; ^*^ *p*<0.05; ^***^ *p*<0.001 denotes the significance levels compared to CYP values. +Mesna represents CYP+Mesna; +QAPF represents CYP+QAPF.

As shown in Fig 3, the i.p. administration of CYP (300 mg/kg) resulted in serious bladder damage, including severe hemorrhage and edema when compared to the saline group, as previously shown [21, 26]. According to the macroscopic evaluation of the bladder, the reference compound Mesna (60 mg/kg) caused a significant inhibition of the hemorrhage (Fig 3A) and of the edema (Fig 3B) induced by CYP. Treatment with QAPF was also able to markedly inhibit the formation of hemorrhage and edema after CYP application (Fig 3A and 3B, respectively).

CYP-induced edema was also evaluated by determining the weight of the empty bladder per 100 g of body weight. Consistently with our findings from the macroscopic evaluation indicating bladder edema, the wet bladder weight was increased after CYP administration (Fig 3C and Table in S1 Table). Regarding the effects of QAPF, the results of the bladder weight assay were in agreement with the data obtained for the reduction of edema (Fig 3B), demonstrating that with 100 mg/kg QAPF, the bladder weights from treated animals were similar to those of animals treated with Mesna (Fig 3C and S1 Table). Furthermore, no change was observed in the bladder of the animals when they were treated with QAPF alone at 20 mg/kg (S1 Table) or at 100 mg/kg (Fig 3A, 3B and 3C). Based on these results, we chose to examine a QAPF dose of 100 mg/kg in the following experiments.

### QAPF partially reduces the histological changes induced by CYP

**Fig 4:**
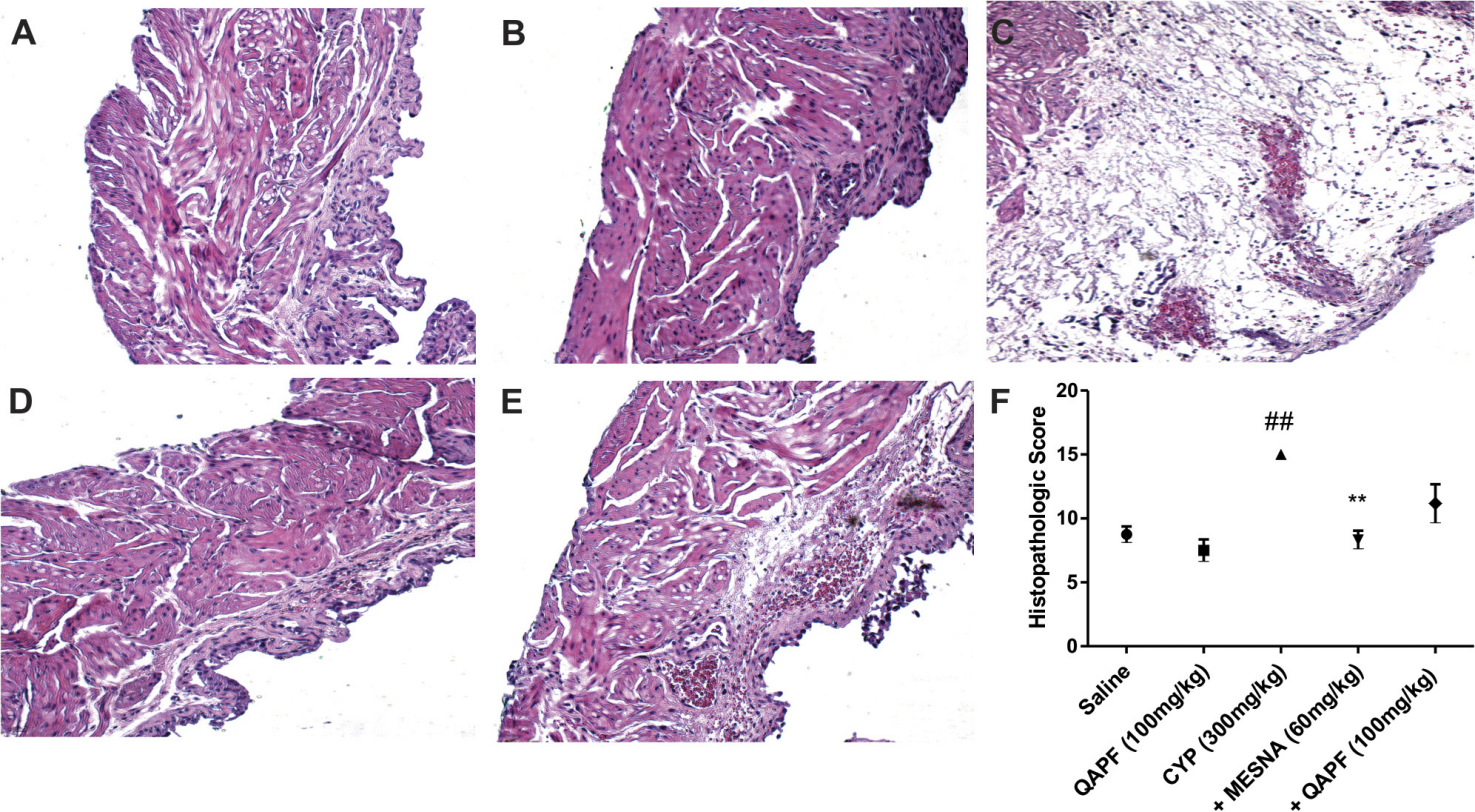
Representative images of histological evaluations using hematoxylin and eosin. (A) Saline groups; (B) animals treated with QAPF only; (C) CYP groups; (D) animals treated with Mesna before and after CYP administration; (E) animals treated with QAPF before and after CYP administration. Original magnification × 40 in all panels. (F) Histopathologic alterations were scored as described in the Materials and Methods. Each column represents the mean of at least 4 animals, and the vertical lines show the SEM. ^##^ *p*<0.01 denotes the significance levels compared to saline values; ^**^ *p*<0.01 denotes the significance levels compared to CYP values. +Mesna represents CYP+Mesna; +QAPF represents CYP+QAPF.

CYP administration resulted in severe bladder damage, including the loss of urothelium, the presence of inflammatory cell infiltration, the presence of much ulceration, fibrosis, a severe degree of edema and hemorrhage (Fig 4C). Treatment with QAPF produced a partial, but not significant, reduction in the histological characteristics of hemorrhagic cystitis. We observed a partial recovery of urothelium, lesser inflammatory cell infiltrate, fewer mucosal ulceration and fibrosis, and mild to moderate edema and hemorrhage (Fig 4E). Animals that were treated with Mesna (60 mg/kg) exhibited characteristics close to those of normal animals, with mild edema and few ulcerations (Fig 4D), resulting in representative histopathological scores (Fig 4F). No significant changes were observed in mice treated with saline (Fig 4A) or QAPF alone (Fig 4B).

### QAPF reduces MPO activity

**Fig 5:**
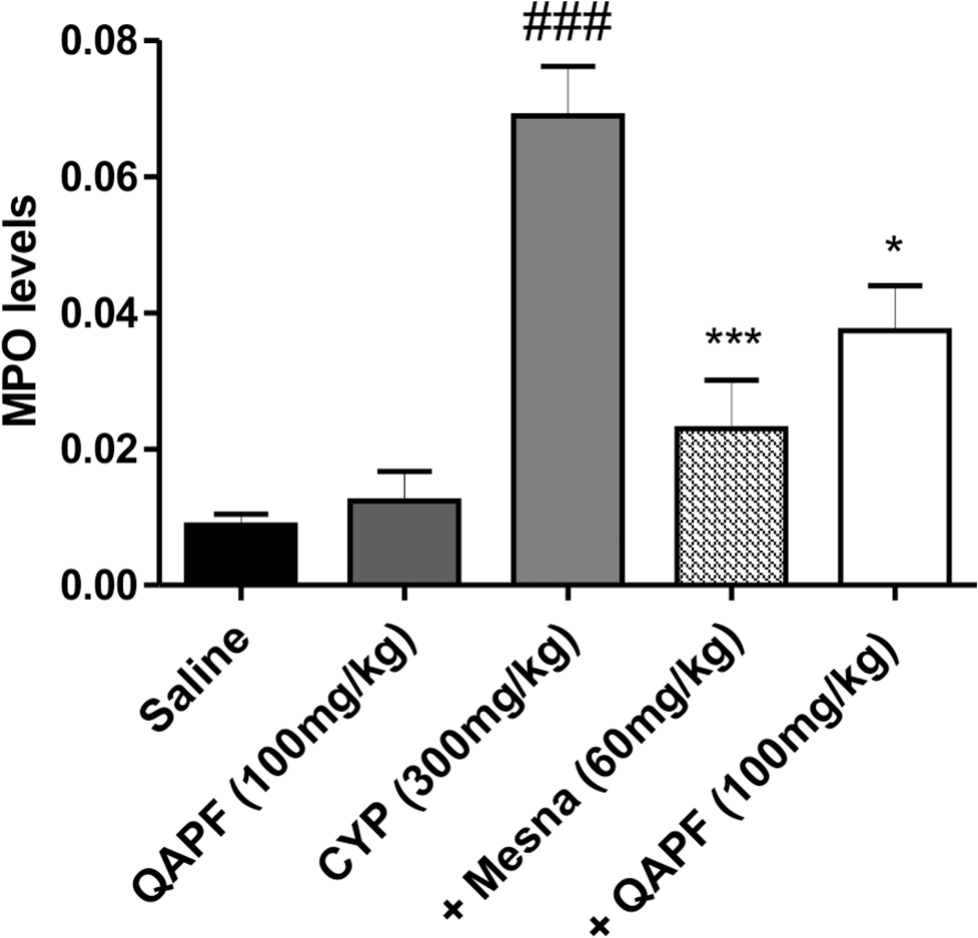
Effect of treatment with Mesna or QAPF from *Uncaria tomentosa* on MPO activity. Each column represents the mean of at least 3 animals, and the vertical lines show the SEM. ^###^ *p*<0.001 denotes the significance levels compared to saline values; ^*^ *p*<0.05; ^***^ *p*<0.001 denotes the significance levels compared to CYP values. +Mesna represents CYP+Mesna; +QAPF represents CYP+QAPF.

The massive migration of neutrophils is also a feature of cystitis induced by CYP administration, as indicated by the increase of MPO activity in the bladder tissue when compared to that of the saline group. This increase of MPO activity levels detected in the bladder tissues of mice treated with CYP was significantly reduced by the treatment with Mesna and QAPF (Fig 5).

### QAPF decreases the cytokine levels of IL-1β in the bladder

**Fig 6:**
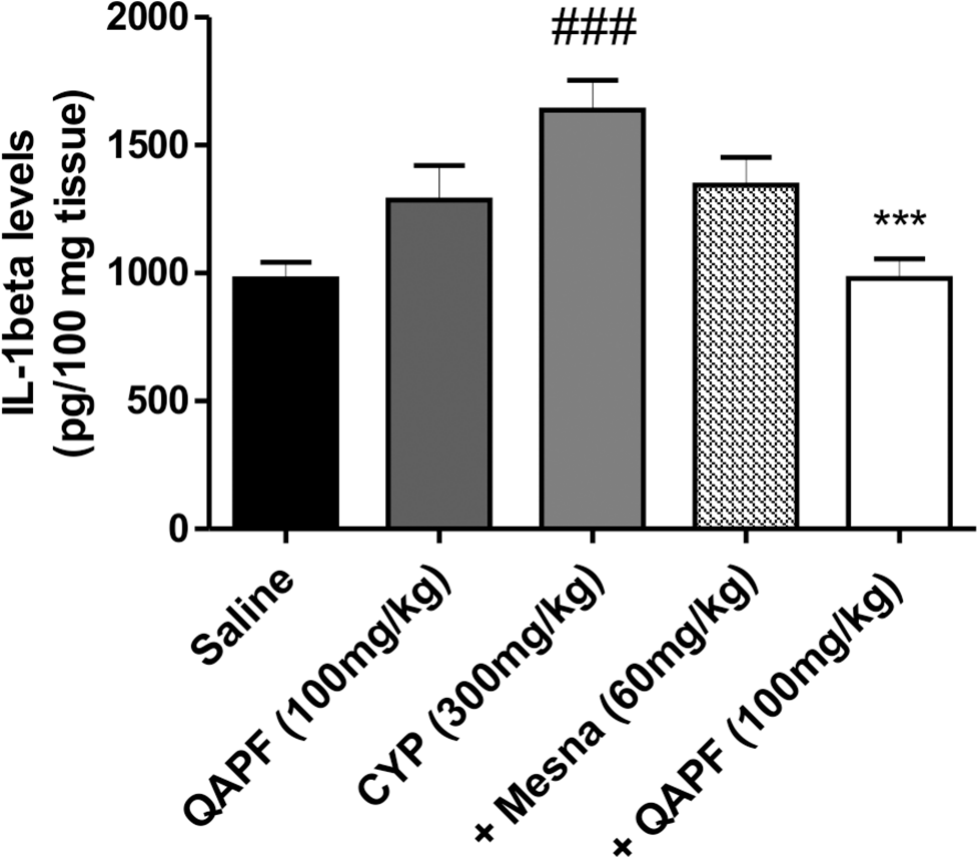
Effect of treatment with Mesna or QAPF from *Uncaria tomentosa* on the generation of IL-1β. Each column represents the mean of at least 8 animals, and the vertical lines show the SEM. ^###^ *p*<0.001 denotes the significance levels compared to saline values; ^***^ *p*<0.001 denotes the significance levels compared to CYP values. +Mesna represents CYP+Mesna; +QAPF represents CYP+QAPF.

The hemorrhagic cystitis induced by CYP administration was also associated with a marked increase of cytokine IL-1β. Levels of IL-1β in the bladder tissue of animals treated with QAPF were significantly lower than those observed in non-treated CYP mice bladders, reaching values that were similar to those measured in the saline group. In this assay, Mesna did not cause any significant effect on IL-1β levels (Fig 6).

### QAPF prevents the increased expression of P2X7 receptors induced by CYP

**Fig 7:**
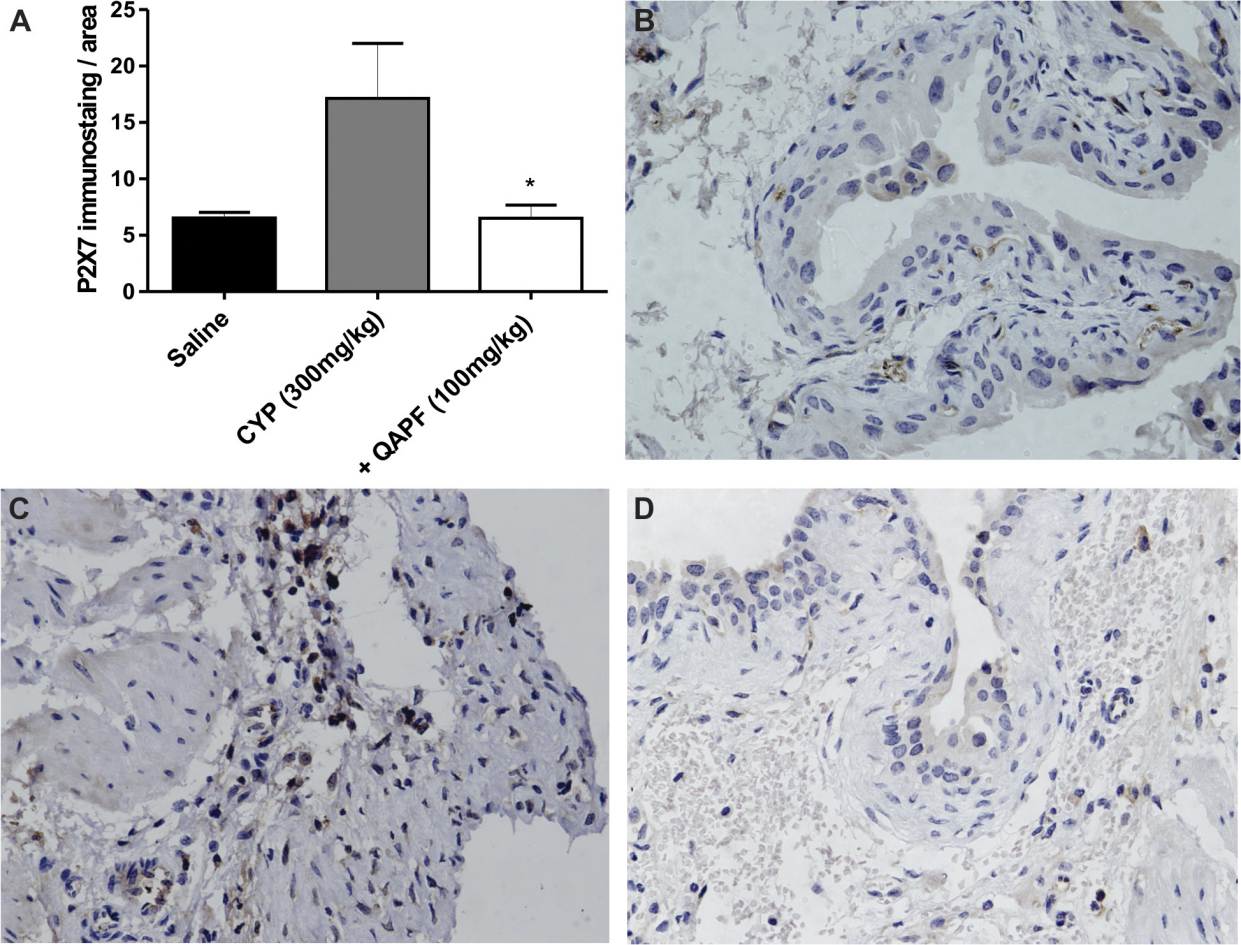
Effect of treatment with QAPF from *Uncaria tomentosa* on P2X7 receptor expression in mouse bladder layers. (A) Each column represents the mean of at least 3 animals, and the vertical lines show the SEM. ^*^ *p*<0.05 denotes the significance levels compared to CYP values. Representative images demonstrate low immunostaining for the P2X7 receptor in animals treated with (B) saline, whereas the immunoreaction for this receptor was markedly enhanced in the bladder submucosal layer of mice treated with (C) CYP; P2X7 was markedly decreased in mice treated with (D) QAPF from *Uncaria tomentosa*. Original magnification ×40 in all panels. +QAPF represents CYP+QAPF.

As shown in Fig 7A, bladder P2X7R expression of CYP-treated animals was increased when compared with that of saline-treated mice. Interestingly, QAPF treatment prevented the increased expression of P2X7R induced by CYP. Fig 7B shows a representative image of P2X7R immunoreactivity in urothelial and submucosal layers of saline-treated mice. The immunoreactivity for P2X7R was markedly increased in the bladder submucosal layer of CYP-treated mice (Fig 7C), whereas the treatment with QAPF promoted a decreasing in the immunoreaction in this layer (Fig 7D). The decreased immunoreaction for P2X7R promoted by QAPF treatment might be due to the diminished neutrophils migration as is indicated by MPO assay (Fig 5). In addition, it is possible to observe that the bladder of mice treated with QAPF presented preserved urothelial cells with immunoreactivity to P2X7R (Fig 7D).

## Discussion

In cases of chronic inflammation and urinary tract diseases, decoctions containing the bark of *Uncaria tomentosa* have been widely used in traditional medicine [1, 2, 3]. These properties attributed to *U*. *tomentosa* encouraged us to investigate the potential effects of its quinovic acid glycosides purified fraction (QAPF), in nociceptive and inflammatory events in a model of hemorrhagic cystitis (HE) induced by cyclophosphamide (CYP) in mice. In this experimental model, widely used to evaluate visceral pain [21, 28], the systemic treatment of mice with QAPF clearly prevented the development of nociceptive behavior induced by CYP (Fig 1) and decreased the visceral sensitivity in the pelvic area (Fig 2) with an efficacy comparable to the reference compound Mesna. Noteworthy, the antinociceptive actions reported to QAPF do not change the general locomotor activity, suggesting that the antinociceptive actions do not appear to involve central effects (S1 Fig). These data suggest the application of QAPF as a potential therapeutic approach to control visceral pain induced by CYP and may justify the traditional medicinal use of *U*. *tomentosa* in relieving inflammation.

CYP-induced toxicity is mainly attributed to the metabolite acrolein that can cause subepithelial edema, ulceration, neovascularization, hemorrhage and necrosis when in direct contact with the urothelium, as previously demonstrated in rodent models [15]. A single administration of CYP causes extensive damage to the urothelium and promotes increases of both edema and hemorrhage [13, 21]. Notably, the pretreatment with the highest dose of QAPF consistently showed a significant reduction of hemorrhage, edema and gross bladder weight in mice, similar to the effects of the reference compound Mesna (Fig 3A, 3B and 3C). These results are consistent with the histological analysis, although in the histological score, the QAPF-treatment partially reduced inflammatory events induced by CYP (Fig 4). The quinovic acid glycosides from *U*. *tomentosa* are considered its most potent anti-inflammatory constituents [8]. Based on this literature data and our experimental results, we conclude that QAPF from *U*. *tomentosa* is a good candidate for further in vivo studies.

It seems that the analgesic effects observed during QAPF treatment may be mediated by the modulation of inflammatory responses. Therefore, the recruitment of neutrophils into the bladder of the mice was measured by myeloperoxidase activity (MPO). In this work, we demonstrated that the increase in MPO activity following CYP administration was markedly attenuated by both Mesna and QAPF (Fig 5). Furthermore, treatment with QAPF significantly decreased the secretion of the cytokine IL-1β to basal levels (Fig 6). However, previous in vitro data revealed that the hydroethanolic extract of *U*. *tomentosa* has the ability to promote IL-1β secretion, via NF-κB inhibition [10, 11]. Based on the results obtained in this work, it is feasible to suggest that part of the antinociceptive and anti-inflammatory action induced by QAPF is likely mediated by a decrease in neutrophils recruitment and a decreased release of IL-1β.

P2X7 receptors are constitutively expressed in the urothelium and submucosal of rat [29, 30] and mouse urinary bladder under normal conditions [21, 31] and are up-regulated in the urinary bladder of patients with symptomatic outlet obstruction [32] and bladders of CYP-induced HE-model mice [21]. Furthermore, P2X7 receptors are involved in neutrophil infiltration and in the maturation and release of the pro-inflammatory cytokine IL-1β [33]. Although the individual inhibition of molecules such as IL-1β has already been demonstrated to decrease bladder damage induced by CYP [34, 35], we decided to investigate the role of QAPF on P2X7R levels due the significant reduction of MPO activity observed in this work. First, we confirmed by immunohistochemical analysis that P2X7R expression is up-regulated approximately three-fold in the bladder submucosal layer in the same model of HE induced by CYP (Fig 7A and 7C), as already demonstrated by our group [21]. Notably, QAPF partially prevented the loss of the urothelium with consequent returning of immunoreactivity to P2X7R in this layer (Fig 7D) as in saline group (Fig 7B). Importantly, we observed a marked down-regulation of P2X7R in the submucosal layer of the bladder (Fig 7A and 7D) after treatment with QAPF. We suggest that this down-regulation is most likely occurring due to the reduced migration of neutrophils, as observed by decreased MPO levels in the inflamed bladders (Fig 5).

Given this set of results we hypothesized that the QAPF, as well as Mesna, could bind to acrolein (CYP toxic metabolite) producing an inactive compound, thereby minimizing toxic effects on the bladder. This hypothesis is plausible because QAPF, by preventing cellular damage (edema and hemorrhage) induced by CYP (Figs 3 and 4), probably decreased the release of several inflammatory mediators, among them nucleotides as ATP, which is a specific P2X7R agonist. As previously demonstrated in the same experimental model, neutrophils and macrophage migration depends of P2X7R activation [21]. As our results clearly indicate, the neutrophils migration to bladder tissue (as seen in diminished MPO activity in Fig 5) as well the release of the cytokine IL-1β (Fig 6) were reduced. The P2X7R immunoreactivity was also reduced in the bladder submucosal layer (Fig 7), suggesting that QAPF prevents the neutrophils migration to this tissue and consequently prevents the up-regulation of P2X7R in this immunological cells. We could therefore hypothesize that QAPF mediated its effects by modulated the inflammatory responses. Therefore, the involvement of P2X7R in the bladder tissue inflammation induced by CYP cannot be excluded since the pharmacological blockade or the genetic deletion of P2X7R prevented the nociceptive and inflammatory events, as we have previously shown [21].

Although more studies are necessary to prove this hypothesis, the results of the present study demonstrated that QAPF from *U*. *tomentosa* exhibits a protective effect on HE-induced urothelial damage and reduces visceral pain, with decreased IL-1β levels and down-regulate P2X7R expression, most likely by inhibit neutrophils migration. Taken together, these results reveal that systemic treatment with QAPF is able to prevent significant nociceptive and inflammatory events associated with HE and may offer a promising therapeutic approach for the prevention and/or adjuvant therapy for treating this disease.

## Supporting Information

S1 Fig Locomotor parameters, assigned at 30-min intervals during 4 h, after the injection period of different treatments in CYP-induced hemorrhagic cystitis in mice. The locomotor parameters were evaluated in the open-field arena.
(TIF)

S1 Table Wet bladder weight analysis in the groups: saline, QAPF, CYP, CYP + Mesna and CYP + QAPF. (XLS)
