# Supplementary figures and images for: The Quinovic Acid Glycosides Purified Fraction from Uncaria tomentosa Protects against Hemorrhagic Cystitis Induced by Cyclophosphamide in Mice
Source: PLoS One. 2015 Jul 8;10(7):e0131882. doi: 10.1371/journal.pone.0131882 (PMC4496084; doi:10.1371/journal.pone.0131882)

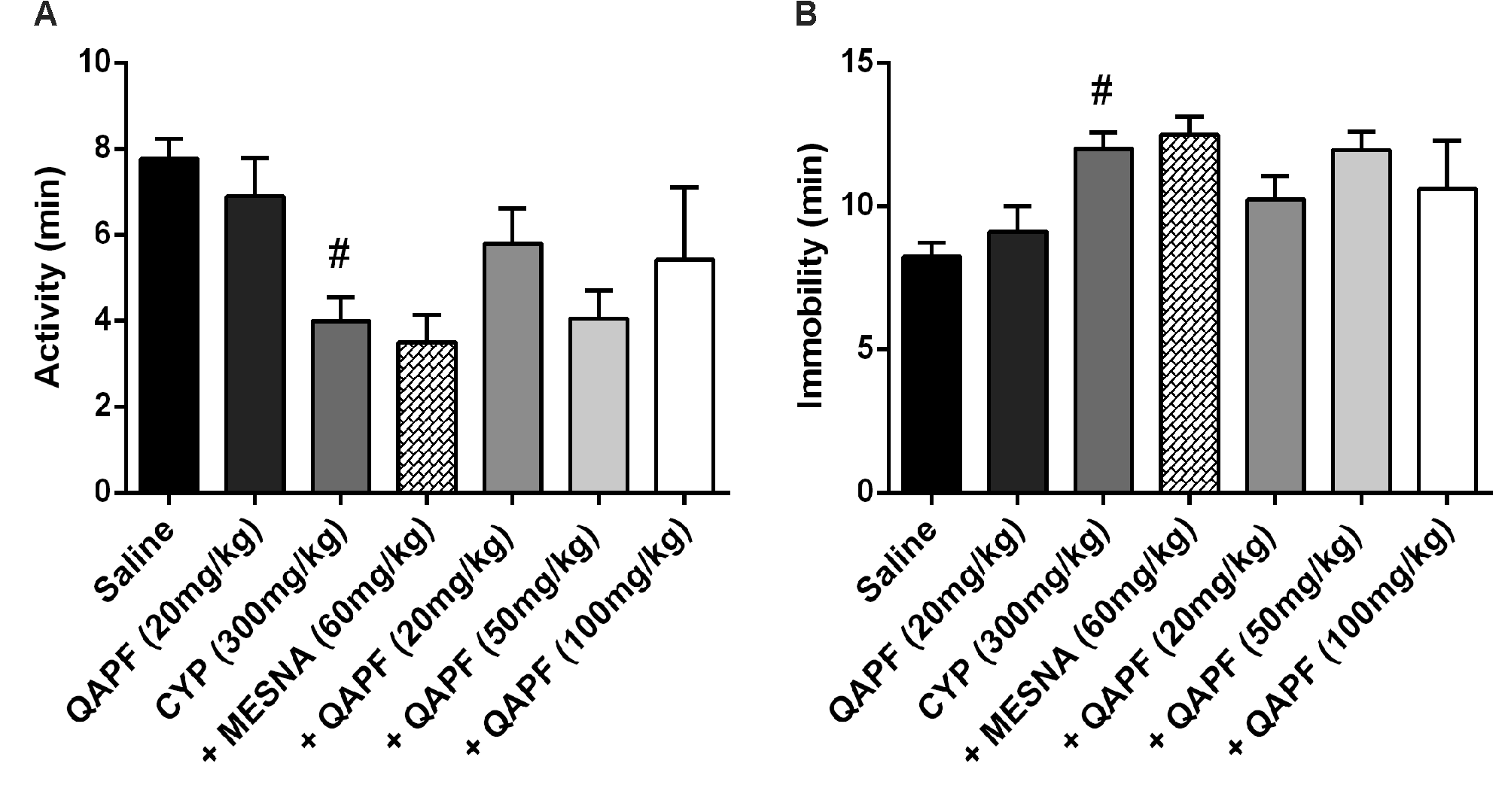

Supplement: S1 Fig — The locomotor parameters were evaluated in the open-field arena. (TIF) [file pone.0131882.s001.tif]
